# Observations of Triple Network Model Connectivity Changes by Functional Magnetic Resonance Imaging in a Single Early-Stage Dementia Participant Pre- and Post-craniosacral Therapy: A Case Report

**DOI:** 10.7759/cureus.97329

**Published:** 2025-11-20

**Authors:** Wei-Che Lin, Ann Wu, Nai-Ching Chen, Belinda Ha

**Affiliations:** 1 Diagnostic Radiology, Kaohsiung Chang Gung Memorial Hospital, Kaohsiung, TWN; 2 Diagnostic Radiology, Chang Gung University College of Medicine, Kaohsiung, TWN; 3 Diagnostic Radiology, Kaohsiung Municipal Fong Shan Hospital, Chang Gung Medical Foundation, Kaohsiung, TWN; 4 Diagnostic Radiology, Kaohsiung Municipal Ta-Tung Hospital, Kaohsiung, TWN; 5 Thyroid Head and Neck Ablation Center, Kaohsiung Chang Gung Memorial Hospital, Kaohsiung, TWN; 6 Medicine, National Sun Yat-Sen University, Kaohsiung, TWN; 7 Craniosacral Therapy, AnAn Wellness, Austin, USA; 8 Neurology, Kaohsiung Chang Gung Memorial Hospital, Kaohsiung, TWN; 9 Clinical Research, AnAn Wellness, Austin, USA

**Keywords:** alzheimer's, craniosacral therapy, dementia, rs-fmri, triple network model

## Abstract

Resting-state functional magnetic resonance imaging (rs-fMRI) is a noninvasive imaging technique that measures spontaneous brain activity to map functional connectivity within and between brain networks characterized as the triple network model (TNM). In Alzheimer's disease (AD), rs-fMRI has been used to detect early network disruptions, track disease progression, and evaluate therapeutic interventions. While craniosacral therapy (CST) has shown clinical benefits for conditions like chronic pain and migraine, its impact on TNM connectivity in AD, as evidenced by rs-fMRI, has not been explored. This case report involves a 79-year-old man with early-stage AD who presented with mild delusions, anxiety, irritability, and nighttime behaviors and a Mini-Mental State Examination (MMSE) score of 24 and a Clinical Dementia Rating (CRD) of 0.5, indicating a mild neurocognitive disorder. Preliminary rs-fMRI data revealed changes in the default mode network (DMN), salience network (SN), and central executive network (CEN) following CST. These changes suggest greater connectivity within the CEN and SN, alongside reduced variability in the DMN following CST. These observations suggest potential reorganization of TNM dynamics. The clinical relevance of these findings remains under evaluation. The observations from this single case report limit the ability to draw definitive conclusions about the impact of CST on TNM connectivity in early-stage AD. A further study is needed to determine if the TNM changes observed by rs-fMRI can be replicated in additional participants and if the changes are correlated with clinical outcomes. Further studies with larger cohorts, extended treatment durations, and longer follow-up periods are needed to explore the potential clinical benefits of CST in this population.

## Introduction

Craniosacral therapy (CST) is a manual therapy developed by Dr. John Upledger and is based on the hypothesis that the craniosacral dura mater and cerebrospinal fluid (CSF) are integrated as they are parts of the craniosacral system. The CST encompasses the Osteopath concept that the body is a dynamic unit that is able to self-regulate and self-correct toward homeostasis and self-healing. It is a light-touch, noninvasive, hands-on palpation technique [1,2]. It has been hypothesized that the CST could also help Alzheimer's disease (AD) patients because it improves sleep and can encourage the flushing of toxins from brain tissues [3]. Studies have shown that sleep quality is strongly linked with AD [4]. A pilot study found that the CST still point technique reduces the agitation in dementia elderly individuals [5].

Resting-state functional magnetic resonance (rs-fMR) images of the triple network model (TNM), which comprises the salience network (SN), central executive network (CEN) or frontoparietal network (FPN), and default mode network (DMN), have been used in many neurological functional connectivity studies. This neurological model always plays a role in almost all, if not all, cognitive functions. It is thought that cognitive transitions are strongly related to AD [6-8]. Studies have revealed that the DMN is active during rest and is associated with internal, self-focused thought [8]. The CEN, a frontal parietal system, is crucial for actively maintaining and manipulating information in working memory, for task-oriented, rule-based problem-solving, and for decision-making in the context of goal-directed behavior [9,10]. The SN integrates sensory, emotional, and cognitive information and functions as a constant dynamic switch between the CEN and DMN. When the SN identifies relevant and important information that requires attention, it acts as a switch between the CEN and the DMN [6].

## Case presentation

The patient is a 79-year-old man, a retired food company chief executive officer (CEO) in Kaohsiung, Taiwan. He is diagnosed with early-stage AD and has mild symptoms of delusions, anxiety, irritability, and nighttime behaviors. His Mini-Mental State Examination (MMSE) [11] score was 24, and his Clinical Dementia Rating (CDR) [12] was 0.5, indicating mild neurocognitive disorder.

The two sets of rs-fMRI and the CST session were conducted back-to-back at the Department of Diagnostic Radiology, Kaohsiung Chang Gung Memorial Hospital, Kaohsiung, Taiwan. The patient was able to walk between the MRI room and the treatment room and was able to reach the treatment table on his own. He underwent the first round of measurement with rs-fMRI, followed by one hour and 15 minutes of CST, and then received the second round of rs-fMRI. 

MRI settings

Resting functional imaging data were acquired via a 3.0 T Siemens MAGNETOM Skyra MRI scanner (Siemens Healthcare, Erlangen, Germany). Resting-state images from 200 contiguous echo planar imaging whole-brain functional scans (repetition time: 2.5 s; echo time: 27 ms; field of view: 220 mm; flip angle: 77°; matrix size: 64×64; thickness: 3.4 mm; scan time: eight minutes and 27 seconds) were collected. During the resting experiment, the scanner room was darkened, and the participant was instructed to relax, with their eyes closed, without falling asleep.

MRI reading methods

Image preprocessing was performed using the Data Processing Assistant for Resting-State fMRI [13] via MATLAB, SPM12, and DPABI. The rs-fMR image preprocessing procedures included slice timing, head motion correction, skull strips, spatial normalization to MNI standard space, low-frequency filtering from 0.01 to 0.08 Hz, and spatial smoothing with an 8 mm full width at half maximum (FWHM) Gaussian kernel. A seed-based analysis was performed to identify five regions of interest (ROIs) for this functional connectivity (FC) study. There were one ROI (posterior cingulate cortex (PCC)) in the DMN, two ROIs (insula; anterior cingulate cortex (ACC)) in the SN, and two ROIs (lateral prefrontal cortex (LPFC); posterior parietal cortex (PPC)) in the CEN.

CST process

This procedure is performed by a therapist who is a Craniosacral Therapy Diplomate-Certified (CST-D) through the Upledger Institute International. The terminologies used in the following paragraph, such as craniosacral rhythm (CSR) evaluation, stillpoints (SPs) [14], respiratory diaphragm release, thoracic inlet release, occipital cranial base release (OCB) without the platform, and fourth ventricle skull area (CV4), are as defined in the Upledger Institute's 10-step protocol [15]. The direction of energy (DOE) technique is further defined in the Direction of Energy article by Dr. John Upledger [16]. Dr. Upledger further defined energy cyst in an article he wrote as "If the energy cannot be dissipated as heat, the body (according to our theory) concentrates and localizes the energy, and somehow encapsulates it as an energy cyst,…" [17]. An energy cyst always feels dense in palpation. In CST, Inner Wisdom (IW) is defined as "the part of you that knows what's needed for healing" [1].

The patient laid supine on the treatment table. The therapist grounded herself and consciously created a safety container with therapeutic presence, whereas the therapist had only the intention to support the patient's inner healing process. The therapist does not have any pretenses to heal the patient. The therapist subsequently obtained permission to touch with IW of the patient. The therapist evaluated CSR and conducted three SPs at the dorsal foot area of both feet to allow the CSR to be more synchronous between the right and left feet. The therapist then performed respiratory diaphragm release, thoracic inlet release, and OCB without the platform and a 5 g spinal traction from the occipital base. During the spinal traction, a structural restriction in the T12-L1-L2 area was identified, and a DOE technique was performed to release the restriction. Then the therapist moved to the cranium area. The therapist directed the therapeutic intention to the circle of Willis and the cranial blood flow. The therapist then focused on the entire cranium area. Since some structural strains were found in the left hind temporal and parietal areas, the therapist facilitated the release of the strains. The therapist moved to focus on the limbic system. In the third ventricle, the ciliary motility of the ependymal cells that line the left side was found to be sluggish, and ciliary motility on the right side was not detectable; thus, they were encouraged and supported until the ciliary movement of the ependymal cells was detectably smooth. The left hippocampus area was found to be weak and sluggish; it was encouraged and supported. The left temporal/insula area had a large energy cyst, so the therapist spent the next 20-30 minutes here, supporting and encouraging these areas to release and heal. The energy cyst felt smaller and less dense. Since much time had been spent at this location, the therapist obtained permission from the patient's IW to move forward to the next area. The therapist facilitated several other releases throughout the cranium and encouraged the astrocytes to strengthen their blood-brain barrier end-feet. The session ended with two cycles of SP at CV4, thanks to the IW and the patient for coming to the session nonverbally. In addition to the silence-releasing signs, the patient provided information that he underwent spinal surgery when younger and complained that the MRI was very noisy. However, he started to relax after respiratory diaphragm release, and he fell asleep during OCB. The words "focused" and "followed" indicate that the palpation focus is directed onto a certain area. The words "encouraged" and "supported" represent intentional support of the patient's self-healing process. An energy cyst could be formed to encapsulate unmetabolized damaged body tissues due to injury, an undissipated foreign energy that, at the time of entry, is not processed completely by the body, a trapped emotional energy, metabolic waste that was not cleaned completely, or anything else that is foreign to the body. With the therapist's help, the body was able to release either the energy, emotion, or toxin that was trapped and encapsulated in a certain area, and that area of the body was able to return to its original shape, form, and function as much as possible afterwards [2,14-18].

Altered functional connectivity between the pre-CST and post-CST treatment

**Figure 1:**
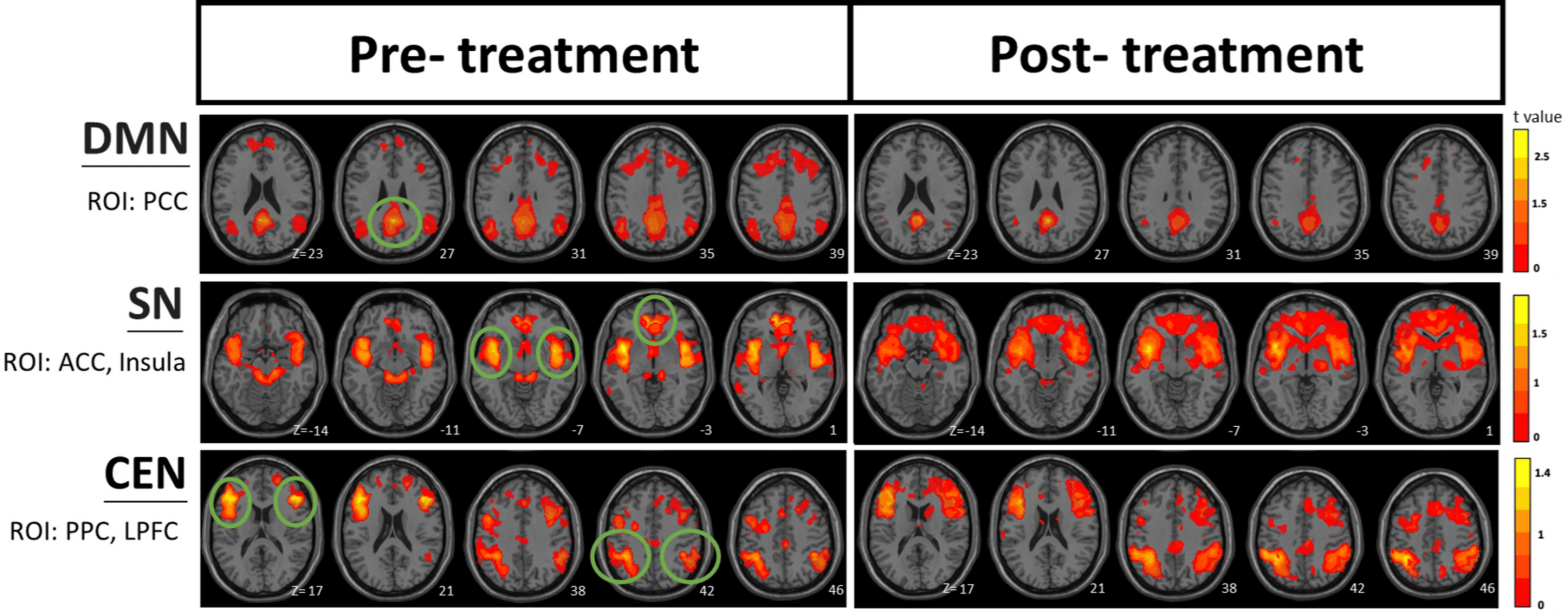
rs-fMR images of DMN, SN, and CEN before and after CST treatment The green circles demark the seed locations of the ROIs: PCC in the DMN, insula and ACC (both left and right sides) in the SN, and LPFC and PPC (both left and right sides) in the CEN. The T-value represents the statistical strength of functional connectivity between the seed region and other brain areas. The Z values do not carry any comparison information here because they are from the same participant. rs-fMR: resting-state functional magnetic resonance; DMN: default mode network; SN: salience network; CEN: central executive network; CST: craniosacral therapy; ROI: region of interest; PCC: posterior cingulate cortex; ACC: anterior cingulate cortex; LPFC: lateral prefrontal cortex; PPC: posterior parietal cortex

Figure 1 provides a visual comparison of the pre- and post-treatment rs-fMRI functional connectivity maps, revealing notable visual differences in the spatial patterns across all three networks. For the DMN, the post-treatment images showed less functional connectivity compared to the pre-treatment images. In contrast, both the SN and the CEN on all transverse axial slices showed more functional connectivity around the ROIs in the post-treatment images.

These visual observations represent qualitative differences in the functional connectivity maps between the two imaging sessions, with the post-treatment patterns showing an apparent shift toward reduced DMN connectivity and expanded SN and CEN connectivity patterns.

## Discussion

In this single-case study, we evaluated pre-CST and immediate post-CST treatment rs-fMR images in an exploratory analysis of potential neuroimaging changes following CST. We utilized the framework of the TNM to analyze and interpret the rs-fMR images. At the time of the CST treatment, the therapist did not have any knowledge of the patient's pre-treatment rs-fMR images. Similarly, at the time of MR image post-processing, the principal investigator was blinded to what the therapist observed and noted during the CST session. 

According to the TNM, these three networks typically operate in a coordinated manner: the SN acts as a switch that alternates between the DMN, associated with internal focus and rest, and the CEN, associated with external attention and cognitive tasks. In healthy individuals, when the CEN is active for task performance, the DMN should be relatively suppressed, and vice versa. The pre-treatment images appeared to suggest simultaneous activity across all three networks (SN, DMN, and CEN), which differs from the typical pattern where DMN and CEN activities are usually inversely correlated [19]. The simultaneous activation pattern could potentially indicate disrupted network switching, though this interpretation remains speculative without quantitative measures. The post-treatment images appeared to suggest a different pattern, with what seemed to be stronger SN connectivity and the typical inverse relationship between DMN and CEN (decreased DMN with increased CEN connectivity). This pattern appeared more consistent with the expected network dynamics described in the TNM. These visual observations could be highly associated with the CST intervention, though other factors could potentially account for these changes, too.

## Conclusions

This exploratory case study documents apparent changes in brain network connectivity patterns observed when comparing rs-fMR images obtained before and immediately after a single CST session.

Several important limitations must be acknowledged. The temporal relationship between CST and the observed changes does not establish causation, as multiple confounding factors may have contributed to the imaging differences. Without quantitative metrics, the observed changes remain qualitative impressions that require statistical validation. A single participant provides insufficient evidence for generalizability or whether any observed changes represent clinical benefit, such as improved brain function or cognitive benefit.

This case study represents the first application of neuroimaging methodology to objectively document brain activity changes temporally associated with CST, providing a basis for future controlled studies to investigate potential neurobiological mechanisms underlying this therapeutic approach. Future studies should employ rigorous study designs, including larger sample sizes, control groups, quantitative analysis methods, and longer-term follow-up assessments, to better isolate the potential effects of CST from other variables and to understand any potential therapeutic effects of CST on brain connectivity in individuals with early dementia or AD.
